# Point of care ultrasound in acute injuries of fingers and thumb

**DOI:** 10.1016/j.tcr.2026.101423

**Published:** 2026-08-11

**Authors:** Zoé Schott, Guillaume Vergnenègre, Sacha Chrosciany, Pascal Kouyoumdjian, Pierre-Sylvain Marcheix, Olivier Mares

**Affiliations:** a Orthopaedics and Traumatology Department, CHRU Limoges, 2, Avenue Martin Luther King, 87042, Limoges, France; b Orthopedic and Traumatology Surgery Department, CHU Nîmes, University Montpellier 1, Nîmes, Place du Professeur Robert Debré, 30029, Nîmes, France

**Keywords:** Ultrasound, POCUS, Finger injury

## Abstract

**Introduction:**

Acute closed trauma to the long fingers and thumb presents a therapeutic challenge. Ultrasound refines clinical examination and enables optimal management. This study aims to assess the role of ultrasound in the management of acute closed trauma of the fingers and the thumb during post-emergency consultations.

**Method:**

Patients were re-evaluated within 15 days of their initial emergency visit. The evaluation combined a clinical examination and a standardized ultrasound assessment. Based on ultrasound findings, initial management was either confirmed or modified. The primary endpoint was the modification of initial management. Secondary endpoints included the evaluating of PRWE-Fr and QuickDASH scores and assessing of changes in the work absence duration.

**Results:**

Twenty-seven patients were evaluated, with a minimum clinical follow-up of three months. The mean age was 33 years. The thumb and the proximal interphalangeal joint were the most frequently affected fingers and joints, respectively. Ultrasound led to modifications in initial management in 77.8% of cases. Return to work was accelerated by an average of 5 days. At three months, mean PRWE-Fr and QuickDASH scores were 12.9 and 9.42, respectively.

**Conclusion:**

Ultrasound is an essential tool in the evaluation and management of acute trauma to the fingers and thumb. It is accessible, easy to perform, and non-irradiating, enhancing the clinical examination by the hand surgeon and enabling early therapeutic adjustments.

**Level of evidence:**

Level III.

## Introduction

Hand injuries are among the most frequent reasons for emergency department visits. Since 2010, their incidence has continued to rise, reaching over 2.1 million cases annually in France, representing a 32% increase in 20 years [1].

Beyond their high frequency, these injuries have a significant socioeconomic impact. They generate substantial healthcare costs and are responsible for many work absences, contributing to absenteeism and reduced productivity [2], [3], [4].

In the emergency department, X-rays remain the main exam for diagnosing fractures, dislocations, and radiopaque foreign bodies. However, it has significant limitations in evaluating soft tissue structures such as tendons, ligaments, pulleys, nerves, and blood vessels [5]. Consequently, capsuloligamentous and tendon injuries, when unaccompanied by radiographic abnormalities, are often underdiagnosed or poorly assessed [6], [7]. Inadequate management can lead to significant functional sequelae, requiring complex secondary surgeries, sometimes resulting in disabilities and hindering the return to work [7], [8], [9], [10], [11], [12], [13], [14].

Ultrasound has become a key tool for hand surgeons. It offers numerous advantages: no radiation, low cost, real-time static and dynamic analysis, bilateral comparative examination, and emergency accessibility [13], [14], [15], [16].

This study evaluates the role of ultrasound in managing acute closed trauma of the fingers and thumb. Our primary hypothesis was that the systematic use of ultrasound would enable optimal adaptation of therapeutic strategies. Secondary objectives included evaluating functional scores (PRWE-Fr [17] and QuickDASH [18]) and the impact of this approach on modifications to work absence durations.

## Materials and methods

This observational, prospective, multicenter pilot study was conducted from January to November 2024. A total of 34 patients aged 18 or older were included after presenting to the emergency department for a closed trauma of the fingers or thumb. Three patients refused to participate in the study, and four did not attend their follow-up consultation.

Upon their initial management in the emergency department, all patients underwent radiographic assessment to rule out the presence of a fracture.

The inclusion criteria were: adult patients presenting to the emergency department following closed trauma to the thumb or long fingers, with no bone injury identified on radiography.

Exclusion criteria included: age under 18, a fracture on the initial X-ray, a wound or skin injury, a congenital malformation, or a pre-existing tumor in the injured finger.

**Fig. 1:**
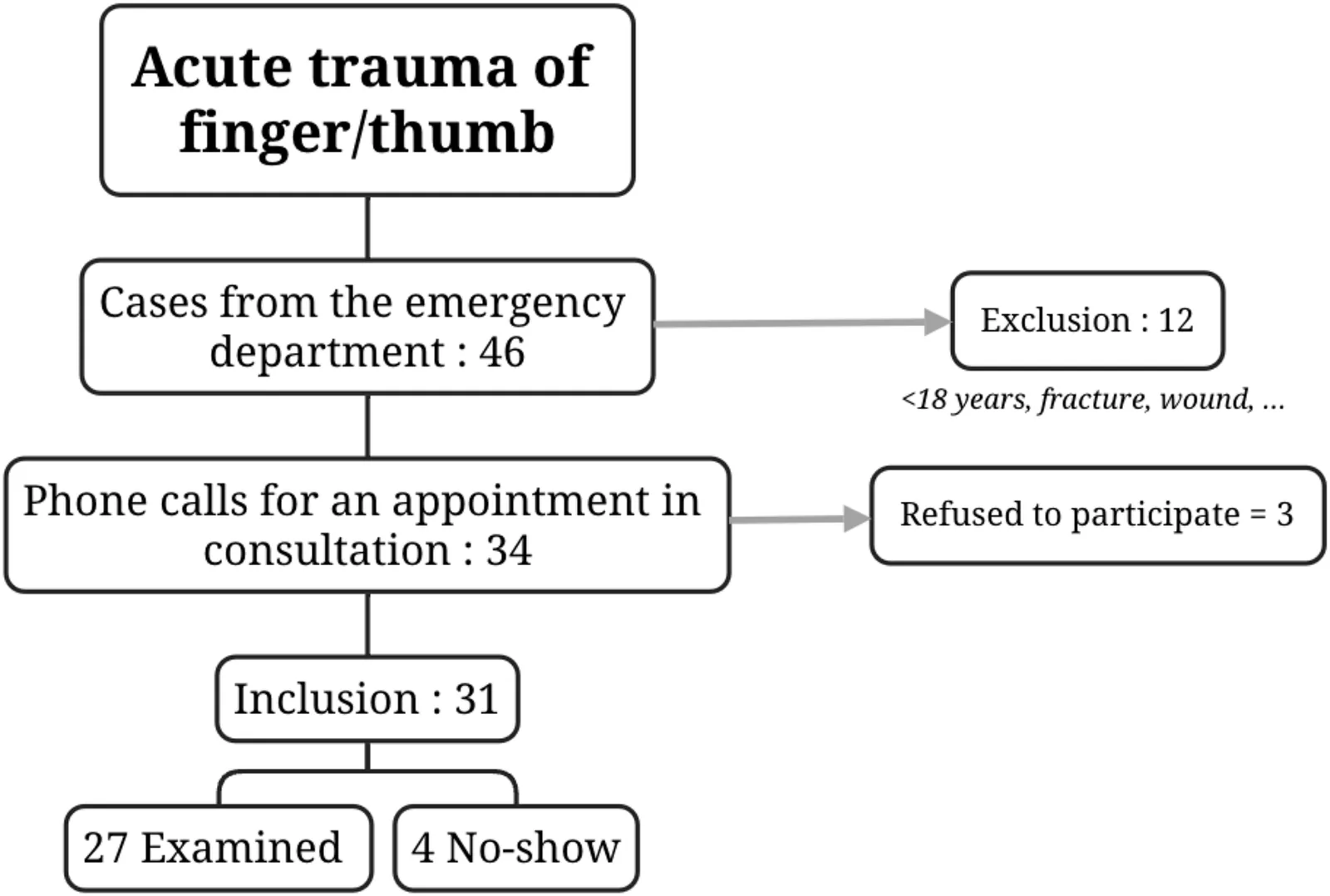
Flow chart.

Within 15 days of the trauma, each included patient was contacted by phone to schedule a follow-up consultation with a surgeon specialized in ultrasound (Fig. 1).

### Clinical examination and ultrasound

The clinical examination assessed pain, bruising, swelling, or joint laxity.

All patients were examined, and a protocolized and standardized ultrasound examination was performed.

Using a musculoskeletal mode, the ultrasound device used was a General Electric Logiq™ P9 XDclear™ with an L8-18i 18 MHz and 24 MHz golf club probe.

For the static examination, the fingers were placed in a neutral position on a towel for the patient's comfort.

### Each structure was examined in both axial and longitudinal planes

**Fig. 2:**
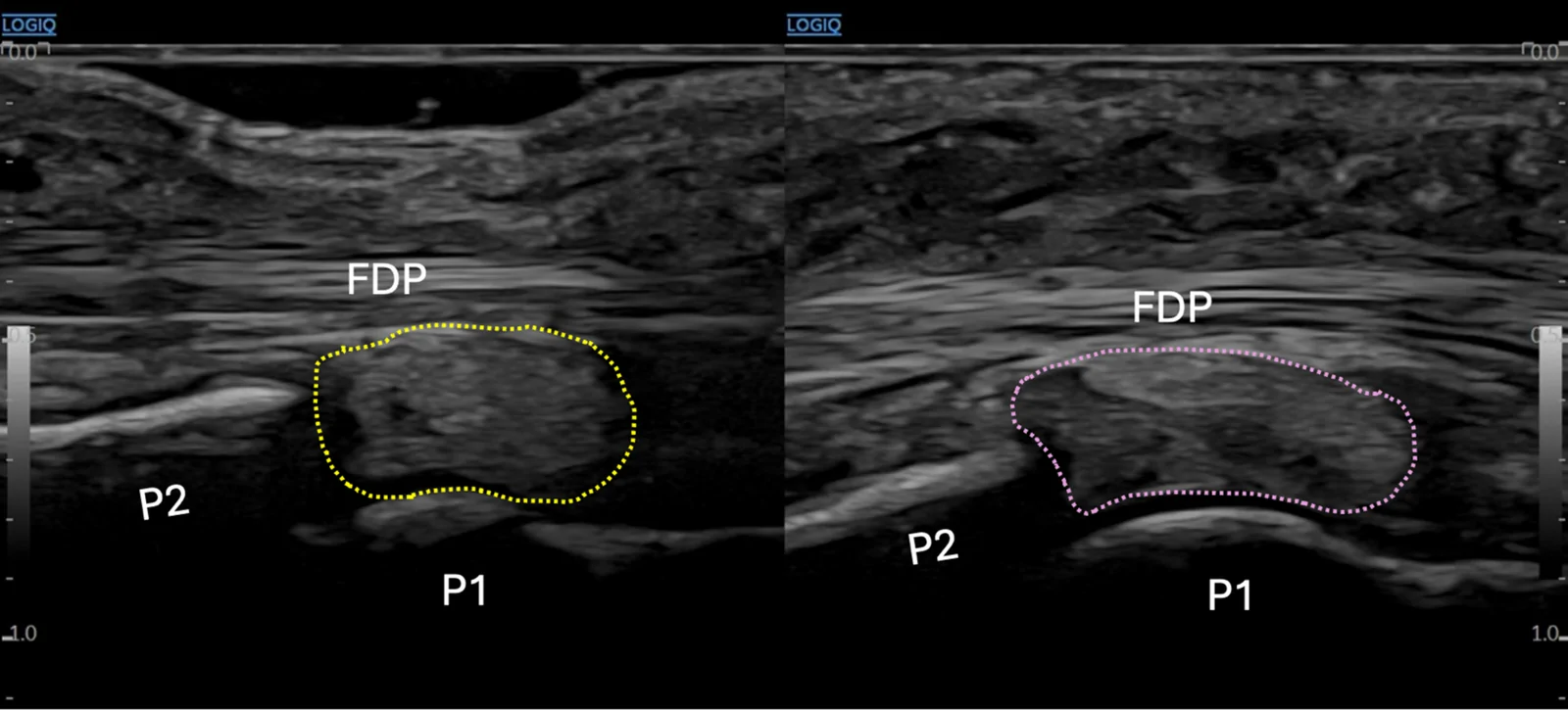
Right image: Volar plate lesion without retraction (pink contouring); Left image: Comparative image of the contralateral finger with normal aspect of the volar plate (yellow contouring); FDP: flexor digitorum profundus; P1: proximal phalanx; P2: intermediate phalanx.

**Fig. 3:**
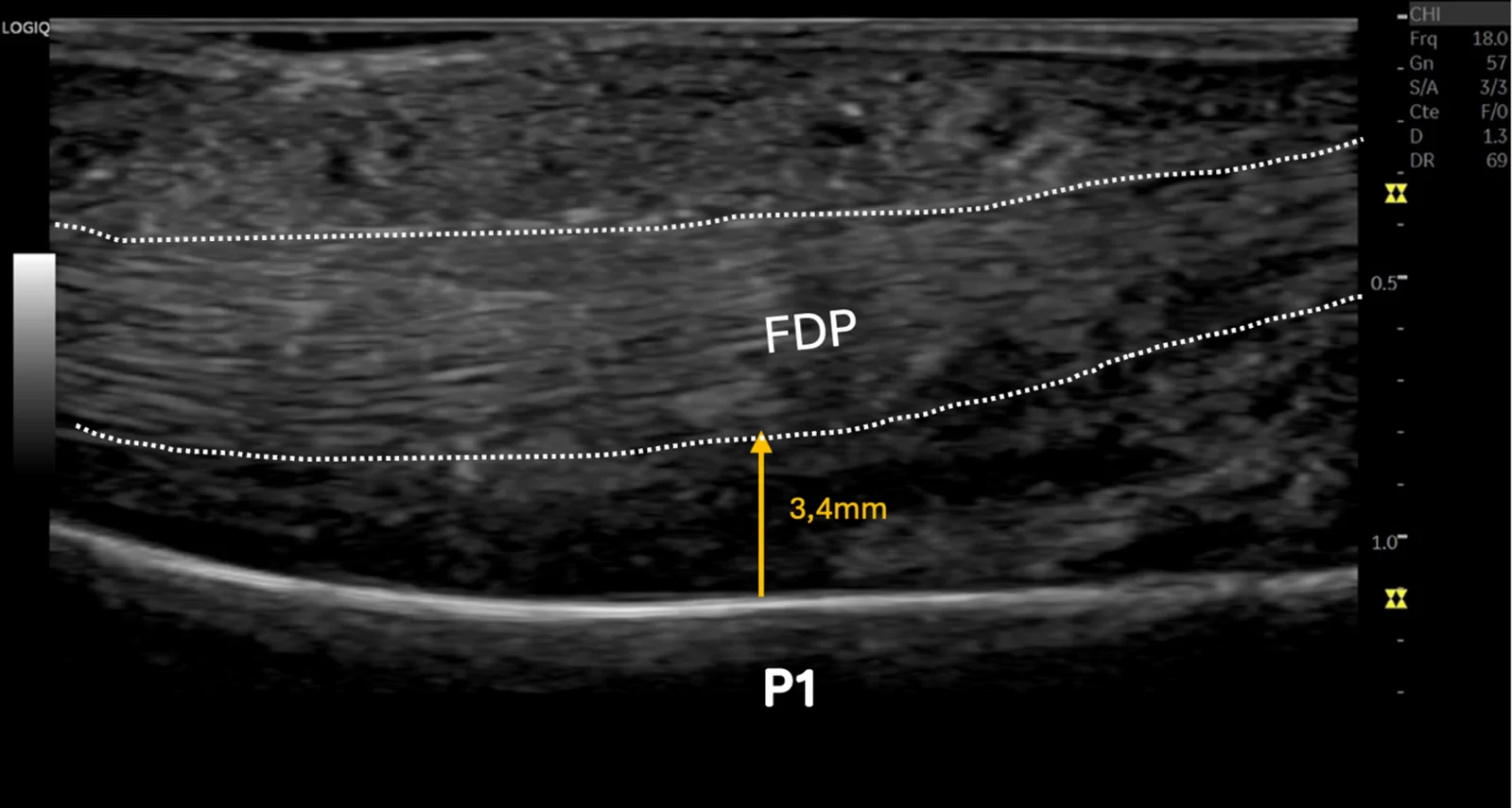
Longitudinal section at the level of the A2 pulley of the middle finger with a tendon-to-phalange distance measured at 3.4 mm (orange arrow); FDP: flexor digitorum profundus; P1: proximal phalanx.

Flexion/extension mobilization was performed passively and actively against resistance for the dynamix examination. The passive hyperextension movement tested the volar plate (Fig. 2). Flexion against resistance evaluated the distance between the phalanx and the flexor tendon to assess pulley injury (Fig. 3).

**Fig. 4:**
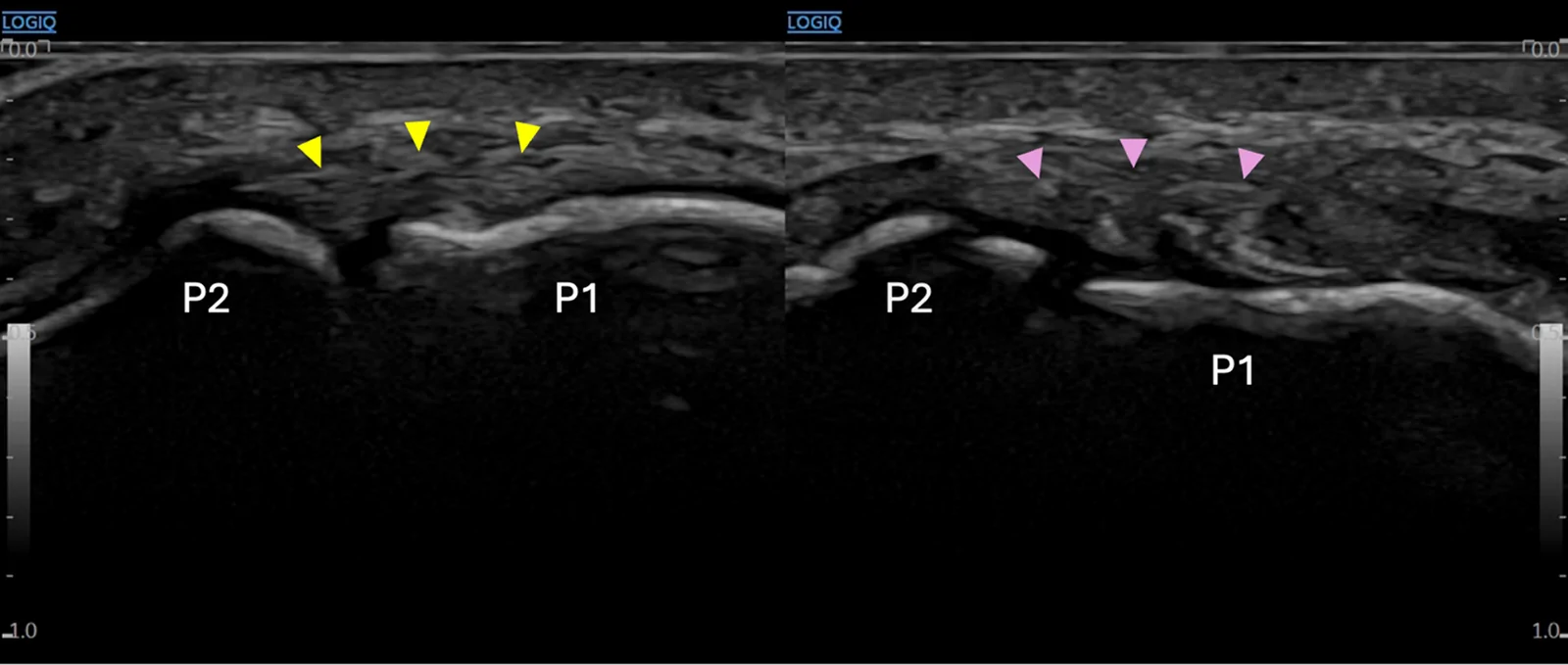
Right image: Avulsion of the radial collateral ligament of the PIP joint of the little finger (pink arrows); Left image: Comparative image of the contralateral finger with normal aspect of the radial collateral ligament (yellow arrows); P1: proximal phalanx; P2: intermediate phalanx.

Valgus and varus testing of the interphalangeal (IP) and metacarpophalangeal (MCP) joints flexed at 30° evaluated the collateral ligaments (Fig. 4).

A Stener lesion was sought after identifying the extensor hood through passive flexion-extension movements of the IP joint for the collateral ligaments of the thumb MCP joint.

A comparative examination was performed on the contralateral or an adjacent finger in case of a suspected lesion.

When the ultrasound probe caused pain, a larger amount of gel or immersion of the hand in a water basin allowed examination without inducing pain.

After each consultation, it was decided whether to modify the initial management or reduce the duration of the work stoppage to allow an earlier return to work.

Three months after the consultation, the patient was contacted again by email or phone to collect functional self-assessment questionnaires: PRWE-Fr and QuickDash, to evaluate the functional outcome of the management implemented following the ultrasound examination.

### Statistical analysis

Quantitative variables were expressed as means ± standard deviation or medians with interquartile ranges, depending on the distribution assessed by the Shapiro-Wilk test. Qualitative variables were presented as percentages and absolute frequencies.

## Results

### Population

**Fig. 5:**
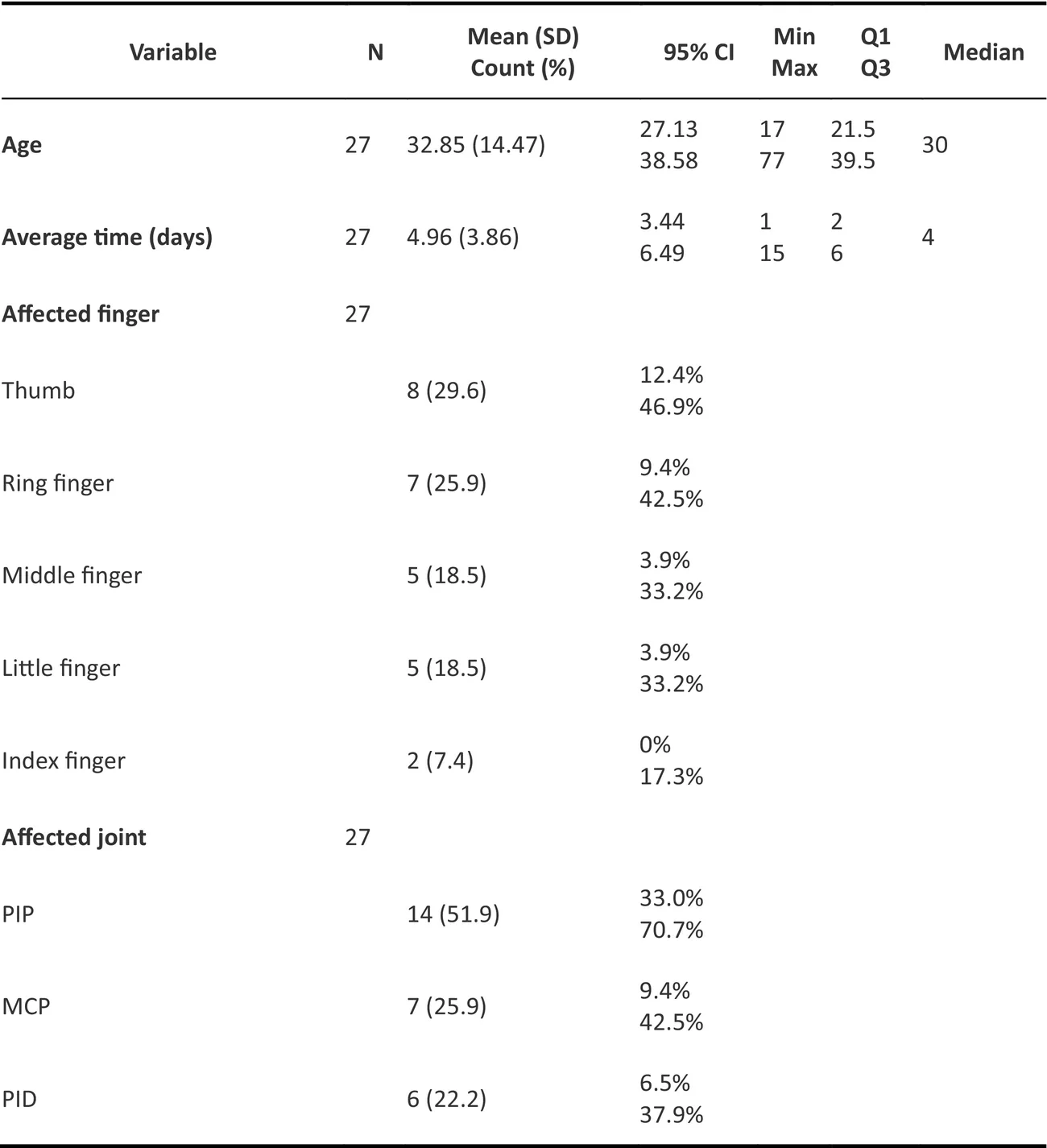
Demographic table.

Our population consisted of 55.6% (*n* = 15) women. The mean age was 33 years (17–77 years). The average time to post-traumatic consultation was 4.96 days, with a median of 4 days (ranging from 1 to 15 days). The dominant hand was affected in 63% of cases (*n* = 17). The most frequently affected finger was the thumb (29.6%) (*n* = 8), followed by the ring finger (25.9%) (*n* = 7), the middle finger and the little finger equally (18.5%) (*n* = 5), and the index finger (7.4%) (*n* = 2). The most affected joint was the proximal interphalangeal joint (51.9%) (*n* = 14), followed by the metacarpophalangeal joint (25.9%) (*n* = 7), and the distal interphalangeal joint (22.2%) (*n* = 6) (Fig. 5).

### Results of ultrasound evaluation

The ultrasound examination identified no lesions in 25.9% of cases (*n* = 7). A lesion of the volar plate was found in 29.6% of cases (*n* = 8). Collateral ligament injuries were observed in 18.5% of cases (5 patients). Sixty percent of collateral ligament injuries involved the ulnar collateral ligament of the thumb (3/5 patients), and a Stener lesion was identified in 1 patient (33%). The extensor apparatus was affected in 14.8% of cases (*n* = 4). Isolated hemarthrosis was found in 7.4% of cases (*n* = 2). An A2 pulley injury was identified in 3.7% of cases (*n* = 1). No flexor tendon injuries were detected.

### Management

Following the post-emergency consultation, initial management was modified in 77.8% of cases (*n* = 21). In 7.4%, this modification involved surgery (n = 2), specifically for ulnar collateral ligament suture of the thumb.

The return to professional activities was, on average, 5 days earlier (SD 7.51 [1.89–8.37]) than initially proposed.

**Fig. 6:**
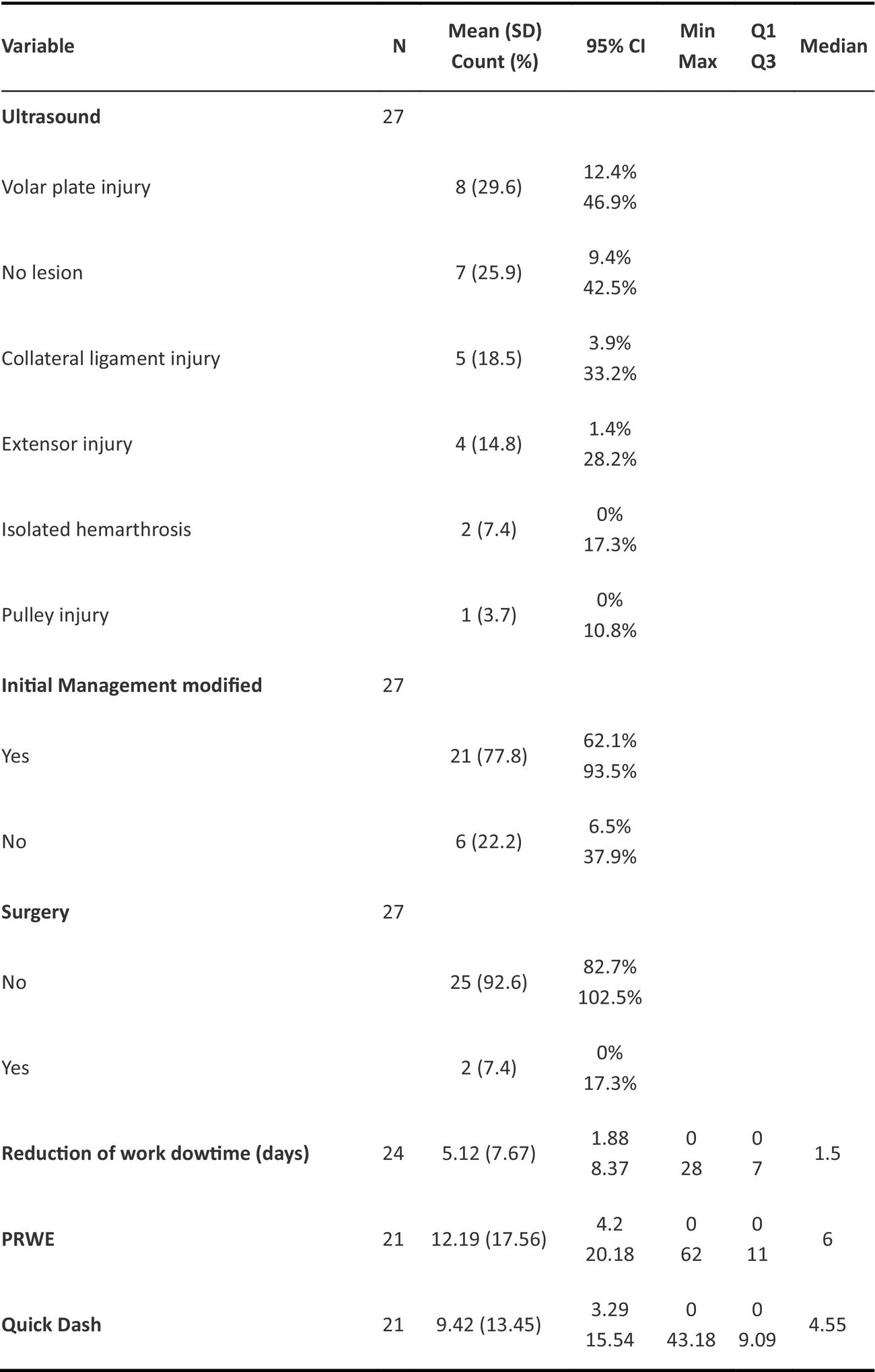
Results.

At the 3-month follow-up, the PRWE-FR score averaged 12.19 (range 4.20–20.18; SD 17.14), and the QuickDash score averaged 9.42 (range 3.30–15.54; SD 13.13) (Fig. 6).

## Discussion

Ultrasound has become an essential tool in managing acute closed injuries of the fingers and the thumb. Its sensitivity and specificity, approaching 100% for tendon injuries [14], [19], [20], represent an essential diagnostic complement.

Our study identified no flexor tendon injuries, confirming their rarity in closed finger injuries.

Conversely, extensor tendons were affected in 14.8% of cases. All the injuries involved the terminal slip, constituting mallet finger lesions. This injury is the most common tendon injury among athletes [21]. In our series, we implemented conservative treatment using a thermoformed dorsal splint for 6 weeks. At the end of the 6 weeks, clinical and ultrasound examinations (both static and dynamic) were performed to ensure proper tendon healing.

Regarding pulley ruptures, Iruretagoiena [22], [23] and Hauger [24] established a clear diagnostic threshold: a bone-to-tendon distance >2 mm is indicative of a complete pulley rupture. In our series, one case of a full A2 pulley rupture was diagnosed in a climber, and orthopedic management using a thermoformed ring allowed a functional recovery, with satisfactory PRWE and QuickDash scores at 3 months (3 and 2.27, respectively).

Early identification of capsuloligamentous and tendon injuries is essential. Without diagnosis and appropriate treatment, these injuries can lead to functional sequelae: digital stiffness, mallet deformity, or joint instability [7], [25], [26].

Fernholm [27] estimates a clinical diagnostic error rate of approximately 30% for osteotendinous injuries of the fingers and hand in Swedish emergency departments.

The most common injuries in our study were lesions of the volar plate (29.6%). In 87.5% of cases, ultrasound modified the initial management, particularly by differentiating injuries according to Wang's classification [13]. Type A and C lesions received appropriate treatment, while two patients with type B injuries declined the proposed surgical management. At 3 months, the average functional scores (PRWE: 11.75, QuickDash: 8.52) reflect satisfactory recovery, although no reference studies are yet available for comparison.

Among ligament injuries, the ulnar collateral ligament of the thumb is the most frequently affected [28]. Three patients in our series had injuries to this ligament, including one case with a Stener lesion requiring surgery. In another case, ultrasound enabled diagnosis when clinical testing was impossible due to pain. This patient received early surgical management, preventing chronic instability.

While MRI is often considered the gold standard for soft tissue evaluation, its accessibility in emergency settings remains limited. In practice, ultrasound offers significant advantages:

- A cost 3.75 times lower than that of MRI [15]
- Superior axial resolution (150 μm compared to 469 μm for standard MRI), allowing finer visualization of structures such as nerves and ligaments [16]
- Dynamic and comparative analysis, which is challenging to achieve with MRI

As Furrer [15] demonstrated, ultrasound influenced our management in several ways: removal or modification of immobilization (with authorization for early mobilization), adaptation of conservative treatment, and, in some cases, surgical indication.

Ultrasound also allowed better management of work stoppage durations. Thirteen patients could return to their professional activities earlier than expected, thus reducing the total duration of work stoppages by 129 days. This optimization represents an estimated savings of €4876.2 for Social Security.

The literature still lacks studies evaluating the economic impact of hand emergencies in France [29].

Still, our results suggest that the systematic integration of ultrasound could improve clinical management and reduce indirect cost related to professional absenteeism.

This study presents several limitations. Regarding the inclusion criteria, we did not include patients presenting with traumatic injuries associated with open wounds, as the ultrasound gel used was not sterile and could have promoted finger infection. We also excluded patients with radiographically identified bone injury, considering that diagnosis and management in these cases were already sufficiently established and that ultrasonography would not provide any additional benefit. Thus, excluding patients with open wounds and fractures resulted in a lower number of included patients than initially anticipated.

In addition, three patients declined to return to the hospital to undergo the ultrasound examination, considering the process too burdensome.

Furthermore, as this was a pilot study, we were unable to establish a control group.

Finally, all examinations were performed by two surgeons specialized in musculoskeletal ultrasonography and ultrasound-guided surgery of the upper limb, making the implementation of a blinded protocol logistically challenging.

## Conclusion

Ultrasound represents an essential tool in managing acute finger and thumb injuries. Accessible, non-irradiating, and cost-effective, it enhances clinical examination by providing a dynamic and precise evaluation of capsuloligamentous and tendinous structures. Its systematic use optimizes diagnosis, reduces the risk of underestimating injuries, and improves patient therapeutic management.

This pilot study provides promising initial findings and a trend toward better therapeutic management through ultrasound for acute closed hand injuries. However, additional studies, involving larger samples are necessary to validate the diagnostic relevance of ultrasound in its use for acute injuries of the fingers and the thumb.

## CRediT authorship contribution statement

**Zoé Schott:** Writing – review & editing, Writing – original draft, Validation, Project administration, Methodology, Conceptualization, Formal analysis, Investigation, Supervision. **Guillaume Vergnenègre:** Conceptualization, Supervision, Methodology, Project administration, Validation, Writing – original draft. **Sacha Chrosciany:** Conceptualization, Writing – original draft. **Pascal Kouyoumdjian:** Methodology. **Pierre-Sylvain Marcheix:** Supervision. **Olivier Mares:** Conceptualization, Formal analysis, Investigation, Methodology, Supervision, Validation, Writing – original draft, Writing – review & editing.

## Declaration of competing interest

None.
